# Maternal High-Fat Diet Controls Offspring Kidney Health and Disease

**DOI:** 10.3390/nu15122698

**Published:** 2023-06-09

**Authors:** Hsi-Yun Liu, Chen-Hao Lee, Chien-Ning Hsu, You-Lin Tain

**Affiliations:** 1 Department of Pediatrics, Kaohsiung Chang Gung Memorial Hospital, Kaohsiung 833, Taiwan; 2 Department of Pharmacy, Kaohsiung Chang Gung Memorial Hospital, Kaohsiung 833, Taiwan; 3 School of Pharmacy, Kaohsiung Medical University, Kaohsiung 807, Taiwan; 4 Institute for Translational Research in Biomedicine, Kaohsiung Chang Gung Memorial Hospital, Kaohsiung 833, Taiwan; 5 College of Medicine, Chang Gung University, Taoyuan 333, Taiwan

**Keywords:** high-fat diet, kidney disease, developmental origins of health and disease (DOHaD), polyunsaturated fatty acid, hypertension, reprogramming, lipid

## Abstract

A balanced diet during gestation is critical for fetal development, and excessive intake of saturated fats during gestation and lactation is related to an increased risk of offspring kidney disease. Emerging evidence indicates that a maternal high-fat diet influences kidney health and disease of the offspring via so-called renal programming. This review summarizes preclinical research documenting the connection between a maternal high-fat diet during gestation and lactation and offspring kidney disease, as well as the molecular mechanisms behind renal programming, and early-life interventions to offset adverse programming processes. Animal models indicate that offspring kidney health can be improved via perinatal polyunsaturated fatty acid supplementation, gut microbiota changes, and modulation of nutrient-sensing signals. These findings reinforce the significance of a balanced maternal diet for the kidney health of offspring.

## 1. Introduction

The public health debate on dietary fat and health has been continuing for more than half a century. Most epidemiological studies have linked high consumption of fats, especially saturated fats, to an increased risk of cardiometabolic disorder [1]. Nevertheless, direct evidence of the benefits of lipid-lowering by altering dietary fat composition is lacking. Although dietary advice recommends lowering the total fat content [2], the types of fats must be taken into consideration. 

The rising incidence of kidney disease is a global public health challenge that influences all age groups [3,4]. As adult kidney disease can originate in early life [5,6], prevention of kidney disease must begin as early as in the fetal or childhood stage [7]. During development, the kidneys can adapt to environmental stimuli through structural or functional alterations, i.e., developmental origins of health and disease (DOHaD) or developmental programming [8,9]. 

The most well-known structural alteration is a low nephron number. As the basic functional unit of the kidney, a reduction in nephrons can have a major impact on renal programming. A low nephron endowment formed in utero can lead to glomerular hyperfiltration and compensatory glomerular hypertrophy, consequently initiating kidney dysfunction and adult kidney disease [10]. Likewise, renal programming of tubular function may also result in renal dysfunction later in life [11]. 

Recently, several factors have been reported to contribute to renal programming, including maternal diseases, improper nutrition, medication use, toxic substance exposure, exogenous stress, and infection [6,8,9]. Maternal nutrition is the key modifiable factor that may be targeted for controlling kidney disease [12,13,14]. Today, excessive dietary saturated fat intake has increased attention directed toward discovering how a high-fat diet increases the risk of developing kidney disease [15,16,17]. 

Considering that precisely monitoring food intake and manipulating the diet of pregnant women is challenging in human research, animal models offer an invaluable tool to control dietary fat composition and discover the molecular pathways participating in developmental programming [18,19,20,21]. Many animal studies have associated maternal high-fat diets with alterations to structure and function in fetal tissues/organs giving rise to various adult diseases in later life [18,19,20,21]. These phenotypes cover hypertension, adipocyte hypertrophy, dyslipidemia, obesity, increased visceral fat mass, hepatic steatosis, and insulin resistance [6,7,8,9,10]. However, kidney disease has received relatively less attention.

Controlling maternal diet can also be advantageous for renal programming [22]. Multiple reports have revealed that developmental programming of adult disease can be reversible through nutritional interventions during pregnancy and lactation by reprogramming [22,23,24]. Although polyunsaturated fatty acids (PUFAs) are shown to have a broad spectrum of health benefits against several diseases including kidney disease [25,26], how maternal PUFA supplementation can help avert offspring kidney disease remains largely unclear.

**Figure 1:**
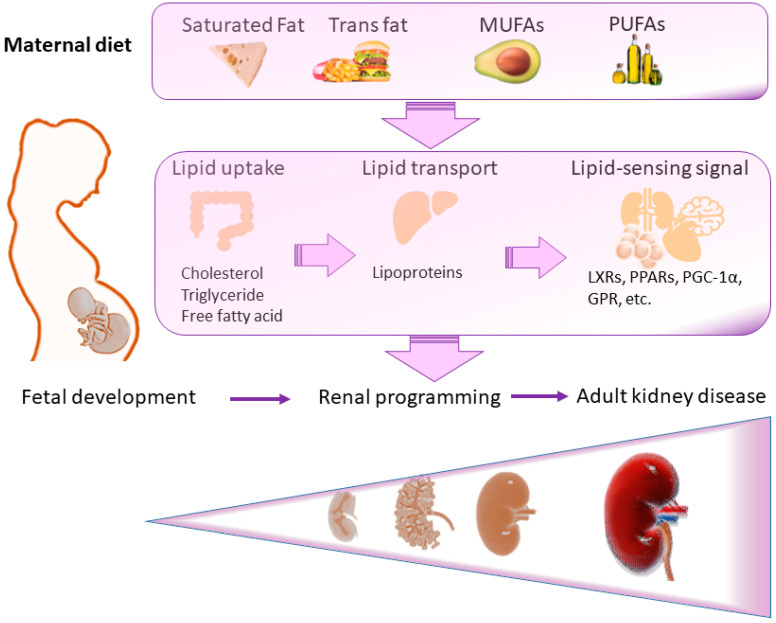
The role of a maternal high-fat diet in offspring kidney health and disease. A maternal diet enriched in saturated fat, trans fats, monounsaturated fatty acids (MUFAs), or polyunsaturated fatty acids (PUFAs) can alter lipid uptake, lipid transport, and lipid-sensing signals in the developing fetus. These changes may cause renal programming, leading to an increased risk for kidney disease in adulthood.

Therefore, this review aims to provide an overview of the roles played by maternal exposure to a high-fat diet in offspring kidney health and disease (Figure 1). Our literature review was performed by searching the databases MEDLINE, Cochrane Library, and Embase using keywords related to a maternal high-fat diet, DOHaD, and kidney disease to survey and establish the evidence in this regard. We used the following search terms: “high-fat diet”, “cholesterol”, “triglyceride”, “fatty acid”, “lipid’, “polyunsaturated fatty acid”, “DOHaD”, “reprogramming”, “developmental programming”, “kidney disease”, “mother”, “pregnancy”, “gestation”, “lactation”, “offspring”, “progeny”, and “hypertension”. We also examined reference lists of articles to detect any extra references that would be related to this review. The last search was made on 30 April 2023.

## 2. Fats in Pregnancy and Kidney Disease

### 2.1. Dietary Fats

Dietary fats are mostly triglycerides [1]. In general, we call the triglycerides in our food “fats” and “oils”. Fats are solid lipids, whereas oils are liquid at room temperature. Fats belong to the triglycerides group, which is a subclass of lipids. The main difference between lipids and fats is that lipids are a broad group of biomolecules, while fats are a type of lipids. 

Fatty acids and glycerol are the building blocks of triglycerides. Dietary fatty acids are categorized into four common types: saturated, monounsaturated, polyunsaturated, and trans fats (Figure 1). Based on carbon chain length (6–24 carbon units) and degree of saturation, fatty acids differ from each other. Saturated fatty acids are saturated with hydrogen with only single bonds, whereas unsaturated fatty acid chains have one (i.e., monounsaturated) or more double bonds (i.e., polyunsaturated) in their carbon chains. The double bonds can be in a cis (same side) or trans (opposite side) position. Naturally occurring fatty acids usually have a cis configuration. By contrast, trans fats are a type of unsaturated fat that originates from artificial or natural sources. Natural trans fats derived from ruminant animals are safe in moderation, but artificial ones may lead to health issues. In addition, polyunsaturated fatty acids (PUFAs) with double bonds that are three carbon atoms (*n*-3; e.g., eicosapentaenoic acid (EPA) and docosahexaenoic acid (DHA)) or six carbons (*n*-6; e.g., arachidonic acid) from the *N*-terminal end of the fatty acid are considered essential fatty acids. 

Various fatty acids have individual biochemical properties and, thus, are able to produce physiological functions. In general, saturated fatty acids and trans fats are linked to an increased risk of cardiovascular disease (CVD). Monounsaturated and polyunsaturated fatty acids are connected with a decreased risk of CVD [27].

In the body, fats confer significant properties on the cell membrane and are mediators of intra- and inter-cellular signaling [16]. In the gut, dietary fats are absorbed, where fatty acids can be esterified with glycerol to produce triglycerides. Cholesteryl esters are generated via the esterification of long-chain fatty acids with cholesterol. 

As cholesterol and triglycerides are insoluble in water, these lipids must be transported by lipoproteins. Plasma lipoproteins can be divided into different classes based on size, lipid composition, and apolipoproteins. Chylomicrons are large triglyceride-rich particles made by the intestine. The removal of triglyceride from chylomicrons by peripheral tissues results in smaller particles known as chylomicron remnants. Very low-density lipoproteins (VLDL) are triglyceride-rich particles made in the liver. VLDL can be further metabolized to low-density lipoprotein (LDL). LDL, when enriched with cholesterol, transports cholesterol to the liver for removal from the organism, whereas the accumulation of oxidatively modified LDL can initiate pathological processes in peripheral tissues. Conversely, high-density lipoprotein (HDL) has cardioprotective properties that operate via reverse cholesterol transport, by which the body removes excess cholesterol from peripheral tissues and delivers them to the liver [28]. HDL particles are enriched with cholesterol.

### 2.2. Fats and Kidney Health

Circulating triglycerides and cholesterol are transported within lipoprotein particles, whereas free fatty acids require albumin as their transporter. Lipids present in the kidneys include triglycerides, cholesterol, free fatty acids, and phospholipids [16]. Renal tubular cells take up circulating free fatty acids disassociated from albumin through specific membrane proteins, for example, fatty-acid-binding protein and fatty acid translocase [29]. These lipids enter the mitochondria, where they are metabolized to yield ATP, thus sustaining energy balance in the tubules [30]. Although lipid metabolism in renal tubular cells protects the kidney against damage under physiological conditions, excess lipid accumulation may cause kidney damage in the tubule cells [17]. 

Prior work has indicated that the risk of the development of chronic kidney disease (CKD) increases with high levels of triglycerides [31], LDL cholesterol (LDL-C) [32], and total cholesterol [33], together with low levels of HDL cholesterol (HDL-C) [34]. Lipid overload and impaired fatty acid β-oxidation (FAO) are able to trigger oxidative stress, inflammation, and renal fibrosis [16]. However, different fatty acids may differentially affect mitochondrial function and kidney health. The saturated fatty acid palmitate has been reported to induce mitochondrial stress and kidney damage, whereas the monounsaturated fatty acid (MUFA) oleate increases FAO, which can protect against saturated fatty-acid-induced kidney damage [35].

Fat accumulation in the kidneys can reduce kidney function in several ways, including impaired renal hemodynamics, increased sodium reabsorption and renin secretion, and activation of the renin-angiotensin-aldosterone system (RAAS) [36]. Increased volumes of perirenal fat might compress the loop of Henle and the vasa recta of the renal medulla, leading to a reduction in tubular flow rate [37]. A reduction in NaCl concentration in the macula densa cells can stimulate renin secretion [38]. Activation of the RAAS, beginning with renin secretion, can further stimulate renal tubular sodium reabsorption. Indeed, fats have a great influence on kidney health and disease [39]. We will not attempt a detailed discussion here, as these observations have been reviewed elsewhere [15,36,37].

### 2.3. Fats and Fetal Development

Maternal diet might alter lipid uptake, lipid transport, and lipid-sensing signals in the developing fetal kidneys, resulting in renal programming (Figure 1). During pregnancy, the fetus requires a significant number of fatty acids and cholesterol. For structural purposes, the fetus needs 1.5 mg of cholesterol per gram of tissue [40]. Fatty acids are required as structural components of tissues, as a source of energy, and as activators of transcription factors [40]. In gestational diabetes, maternal plasma fatty acid levels correlate with fetal lipids, fetal growth, and fat mass [41]. In addition, impaired placental transfer of lipophilic compounds has been shown to be related to intrauterine growth restriction [42]. These observations suggest that lipid metabolism during pregnancy has a role in fetal growth and development [42].

Lipid metabolism involves the uptake of lipids in the gut, the synthesis and degradation of lipids in cells, and transport to compartments such as mitochondria. Phosphoinositides are regulators of key sub-cellular processes including cytoskeletal function, membrane transport, and plasma membrane signaling. The kidney relies on phosphoinositides for physiological processes, such as filtration, solute reabsorption, cell polarization, and signal transduction [43]. It is known that mutations of the genes encoding the phosphoinositide system in the kidney very often result in human genetic kidney diseases, such as Joubert syndrome and Lowe syndrome [43]. Nevertheless, no information is available regarding their impact on renal programming. 

Several lipid-sensing nuclear receptors, including peroxisome-proliferator-activated receptors (PPARs), liver X receptors (LXRs), and PPARγ coactivator-1α (PGC-1α), influence all aspects of lipid metabolism [44]. Previously, our data demonstrated that several PPAR target genes are involved in renal programming and hypertension, such as *Ren*, *Nrf2*, *Sod2*, *Nos2*, *Nos3*, *Sirt7*, and *Sgk1* [45]. Since PPARs play a critical role in the pathophysiology of kidney disease [46], it is possible that dysregulated lipid sensing induced by a maternal high-fat diet, such as through the dysregulation of PPARs, may have a close link to renal programming. 

Several lines of evidence support the hypothesis that a maternal high-fat diet might be involved in the pathogenesis of renal programming. First, a previous study revealed that high-fat-intake-induced renal injury is related to a decrease in renal Pax2 expression [47]. Our prior work indicated that several nephrogenesis genes related to reduced nephron numbers are PPAR target genes—for example, *Pax2* [45]. Second, PPARγ was reported to directly regulate a vast array of genes involved in oxidative stress, including *Nos2*, *Nos3*, *Sod2*, and *Nrf2* [48]. Emerging evidence supports the hypothesis that oxidative stress has a critical role in renal programming [6,8,9]; we will discuss this in detail later. Third, it has been observed that several PPAR target genes are RAAS components or sodium transporters. PPARγ has been reported to stimulate renin gene expression [49] and to increase sodium hydrogen exchanger-3 (NHE3) [50]. 

Free fatty acids are ligands for G-protein-coupled receptors (GPR), which are also referred to as free fatty acid receptors (FFAR) [51]. Short-chain fatty acids (SCFAs) are generated from dietary fiber through fermentation via gut microbes and mainly contain acetate, butyrate, and propionate [51]. SCFAs are capable of activating GPR41 and GPR43, whereas long-chain fatty acids can activate GPR40 and GPR120. SCFAs and their receptors play an important role in maternal metabolism and fetal programming [52]. As lipid signaling has been related to fetal programming, it is increasingly important to better identify the actions of maternal exposure to a high-fat diet on lipid signaling and to have the ability to identify mechanisms underlying renal programming. 

Although previous studies reported alterations in kidney structure, i.e., reduced nephron numbers, in offspring exposed to nutritional imbalance during pregnancy and lactation [6], current literature offers little or no understanding of this mechanism in a maternal high-fat diet. However, prior investigations support the hypothesis that a maternal high-fat diet affects the offspring’s renal transcriptome. Aberrant gene expression of several molecular pathways (e.g., PPARs) in the developing kidney may contribute to nephron deficit, dysregulated RAAS, increased sodium transporters, and increased BP [12,13]. All of these mechanisms underlying renal programming are deleterious to future kidney health.

## 3. Renal Programming: The Impact of a Maternal High-Fat Diet

Currently, little reliable information is available regarding whether high fat intake during gestation and lactation leads to adulthood kidney disease in humans. Most pooled epidemiological studies that recruit diverse participants, along with investigating different types of fats from various dietary sources, carry a high possibility of diluting any real findings. Therefore, animal models provide a means to understand the underlying mechanisms of maternal high-fat-diet-induced programming effects. 

Although a high-fat diet has long been recognized as a cause of obesity and related disorders in animal models [18,19,20,21] and the term is in frequent usage, it lacks a precise definition. Different high-fat diets with fat proportions ranging from 20 to 60% energy as fat have been developed in animal models. Additionally, the fat component can vary from animal-derived fats (e.g., butter or lard) to plant oils (e.g., corn or coconut oil) [18,19,20,21,53,54]. Moreover, diets rich in saturated or unsaturated fats might have different impacts on health risks [1]. As this dietary intervention is not standardized, phenotypes induced by a maternal high-fat diet may differ markedly among various animal studies [53,54].

Several animal species have been used to elucidate the effect of a maternal high-fat diet on the progeny, covering non-human primates [55], pigs [56], rabbits [57], rats [20], and mice [20]. As we and others reviewed elsewhere [16,19,31,32], offspring exposed to a maternal high-fat diet may have altered feeding habits, effects on their body composition, reduced cognitive development, increased risk of type 2 diabetes, obesity, insulin resistance, liver steatosis, dyslipidemia, hypertension, etc. 

Although a growing number of animal studies have been reported to determine the impact of a maternal high-fat diet on offspring outcomes [20,21,58,59], only a few studies evaluated renal programming. In this review, we only considered studies restricting exposure to cover the period of nephrogenesis. In rodents, kidney development is roughly from mid-pregnancy to mid-lactation. Table 1 summarizes preclinical studies recording offsprings’ renal outcomes in which maternal high-fat diets were applied during gestation and lactation [60,61,62,63,64,65,66,67,68,69,70,71,72,73,74,75]. Although maternal obesity is frequently studied using rodents on high-fat diets, it is clear that the programming effects of maternal obesity and high fat consumption on offspring outcomes are different [76]. Studies in which high-fat diets were fed to rodents to induce maternal obesity usually started the diets at 4–9 weeks before pregnancy. 

Table 1 shows maternal high-fat diets with different fat proportions ranging from 20 to 58% energy as fat, which is in good agreement with previous studies [18]. However, the high-fat diets used most frequently with rodents did not closely match Western diets, as the latter are lower in fats and protein [76]. In addition to purified high-fat diets, the utilization of a human Western diet, a Western-style diet, or a cafeteria diet has been conducted for metabolic diseases [77,78]. However, none has been applied to study renal programming in this regard.

Rats and mice are the most frequently used species. Adverse renal outcomes in offspring are mainly induced by a maternal diet enriched with saturated fat, such as lard, palm oil, and coconut oil. As presented in Table 1, the influence of a maternal high-fat diet on rat offspring was evaluated from the age of 9 weeks to 6 months. The rodent ages in Table 1 correspond to human ages from adolescence to young adulthood [79]. These renal-programming-related phenotypes cover tubular dysfunction [60,62,65], renal hypertrophy [65], renal function impairment [67,69], proteinuria [67,68,69], renal fibrosis [68,69], and hypertension [70,71,72,73,74,75]. Notably, maternal high-fat-diet-induced renal phenotypes vary, mostly according to age, species, and varied fatty acid fractions and compositions. 

Of note is that kidney disease can be attributed to multiple “hits” [80]. As reported in the DOHaD research, lifelong health can be adversely affected by a series of “hits” experienced at critical developmental periods and across the lifespan [81]. “First hits” are adverse insults experienced by the mother that make the offspring more vulnerable to adult disease. Postnatal insults then present “second hits”, through which prenatally primed vulnerability can be triggered or exacerbated. In some studies, a maternal high-fat diet was applied as the first hit, followed by a second hit to induce kidney disease in later life. For instance, animal models of a maternal and postnatal high-fat diet [82] and a combined maternal high-fat, high-sucrose, and high-salt diet [83] have been used to study renal programming. Another hit may trigger the same programming mechanisms and amplify adverse actions culminating in a disease state. Together, animal models with various types of maternal high-fat diets support the hypothesis that such diets have programming effects on the kidneys of the offspring. 

## 4. Mechanisms Linking Maternal High-Fat Diets to Renal Programming

**Figure 2:**
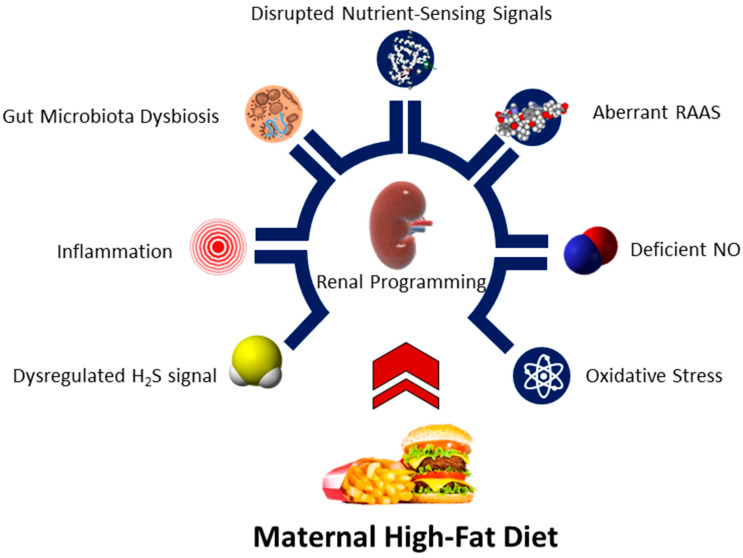
Schematic diagram of the mechanistic links between a maternal high-fat diet and renal programming. NO = nitric oxide. RAAS = renin-angiotensin-aldosterone system. H_2_S = hydrogen sulfide.

To date, several hypothetical mechanisms have been reported to be bound up with renal programming [6,8,9,12]. Among them, oxidative stress, deficient nitric oxide (NO), aberrant activation of the RAAS, disrupted nutrient-sensing signals, dysbiotic gut microbiota, inflammation, and dysregulated hydrogen sulfide (H_2_S) signaling are interrelated with maternal exposure to a high-fat diet and will be discussed in turn (Figure 2).

### 4.1. Oxidative Stress

Oxidative stress, an imbalance between pro- and antioxidant capacity, has been implicated in renal programming [84]. During pregnancy, the developing kidney is vulnerable to overproduction of reactive oxygen species (ROS) under suboptimal intrauterine conditions owing to the deficient antioxidant capacity in the fetus [85]. As we reviewed elsewhere [84], multiple animal models indicated various maternal insults can induce oxidative-stress-related renal programming. 

The mechanistic linking of oxidative stress to renal programming induced by various types of maternal insults covers increased production of ROS [86], decreased antioxidant capabilities [87], increased lipid peroxidation [88], and increased oxidative damage [89]. Conversely, natural and synthetic antioxidants can serve as reprogramming therapies for kidney diseases of developmental origins [90,91,92].

Table 1 demonstrates that maternal-high-fat-diet-primed renal programming is associated with reduced antioxidant activity [63], increased lipid peroxidation [61,63], and increased oxidative DNA damage [61,68,71,72]. A commonly used marker of DNA damage, 8-hydroxydeoxyguanosine (8-OHdG), has been utilized to identify oxidative damage and has revealed such damage to be augmented in the kidneys of adult rat progeny from dams fed on a diet rich in saturated fats [61,68,71,72]. Although various antioxidants show a potential role for reducing oxidative stress in preventing kidney disease [93], their effects on maternal-high-fat-diet-primed renal programming roles are still largely unclear.

### 4.2. Deficient NO

In the kidney, NO carries out important physiological and signaling functions, whereas deficient NO implicates the pathogenesis of kidney diseases [94,95]. During pregnancy, NO has a significant role in the regulation of fetoplacental circulation and fetal development [96]. Deficient NO is one of the mechanistic pathways behind renal programming, whereas perinatal use of NO-based interventions has shown benefits which protect against the developmental programming of kidney disease [81]. Asymmetric dimethylarginine (ADMA) is an endogenous inhibitor of NO synthase, which competes with l-arginine to decrease NO production [97]. ADMA can cause a nephron deficit in cultured rat embryonic kidneys and alter renal transcriptome [98]. Whether ADMA–NO imbalance contributes to the developmental programming of kidney disease remains to be investigated further [98]. 

Deficient NO in kidneys [71,72,75] is related to maternal-high-fat-diet-primed renal programming. High fat intake during gestation and lactation results in decreases in plasma l-arginine concentrations and in the l-arginine-to-ADMA ratio, an index of NO bioavailability [75]. One study demonstrated that maternal bisphenol A exposure (BPA) exacerbates maternal high-fat diet-primed hypertension in adult male offspring, which is associated with increased ADMA concentration and a decreased ratio of l-arginine-to-ADMA [99]. In addition, there was a synergistic effect of maternal high-fat diet and BPA exposure on inducing oxidative damage in offspring kidneys. Conversely, resveratrol, a polyphenolic antioxidant, protected adult offspring from a maternal high-fat diet as well as from BPA-induced hypertension and oxidative damage. The protective action of resveratrol is related to the restoration of NO bioavailability [99] Another study using a dexamethasone and high-fat diet two-hit model demonstrated that adult offspring developed hypertension and kidney oxidative damage that coincided with increases in plasma ADMA and decreases in plasma l-arginine-to-ADMA ratios [100]. Maternal antioxidant therapy by *N*-acetylcysteine could prevent adult offsprings’ hypertension and oxidative damage via restoration of the ADMA–NO balance [100].

As several currently available prescription drugs have the ability to restore the balance of the ADMA–NO pathway [97], additional work is needed to understand the reprogramming actions of NO-based intervention in maternal-high-fat-diet-primed renal programming.

### 4.3. Aberrant RAAS

The RAAS is a key hormone cascade regulating BP and the renal system [101]. There are two RAAS pathways: the classic and the non-classic systems. The classic RAAS is composed of angiotensin-converting enzyme (ACE), Ang II, and Ang type 1 receptor (AT1R). On the other hand, the ACE2–angiotensin (1–7)–Mas receptor pathway is a counter-regulatory RAAS system to offset the harmful effects of Ang II signaling. One such example is that administration of ACE2 activator or ANG-(1–7) during pregnancy has been reported to attenuate hypertension and kidney fibrosis in adult SHR offspring [102].

Activation of the classic RAAS through high fat intake can lead to vasoconstriction, oxidative stress, and inflammation, resulting in kidney disease [103,104]. Hypertension in maternal-high-fat-diet-primed offspring coincides with aberrant activation of the classic RAAS, represented by increases in the renal protein level of AT1R and mRNA expression of *Agt* and *Ace* [64]. 

Glucose transporter 4 (GLUT4) mediates the uptake of glucose [105]. Ang II can mediate GLUT4, which has a role in insulin resistance and in the development of diabetic kidney disease [106]. Prior research revealed that GLUT4 heterozygous (GLTU4 +/−) mice exhibited insulin resistance [107]. In this GLUT4 +/- mice model, maternal high-fat-diet-induced hypertension in offspring was accompanied by increased renal gene expression of renin and the AT1R [66]. Likewise, another study showed maternal high-fat diet increased renal protein levels of AT1R, as well as mRNA expression of *Agt* and *Ace*, in adult rat offspring at 16 weeks of age [108]. Moreover, the non-classic RAAS also participates in renal programming. Another study reported that 16-week-old male rats that are perinatally exposed to a high-fat diet have low Ang-(1–7) levels [109]. ACE2-deficient mice, with low Ang-(1–7) levels, developed hypertension and kidney injury [110]. In the context of experimental kidney diseases, most studies have proposed that the ACE2–angiotensin (1–7)–Mas axis has a protective role [111]. Whether a maternal high-fat diet downregulating the ACE2–angiotensin (1–7)–Mas axis contributes to kidney disease later in life awaits further investigation.

Emerging evidence supports the hypothesis that there is a transient biphasic response with the downregulation of the classic RAAS system in the neonatal period that returns to normal with age [112,113]. A maternal high-fat diet may disrupt this normalization in the adult offspring; thereafter, the classic RAAS system is abnormally activated, whereas the non-classic RAAS axis is downregulated. Considering that renal programming induced by a maternal high-fat diet coincides with aberrant RAAS, it is interesting to elucidate whether targeting RAAS could serve as a reprogramming approach in this regard.

### 4.4. Disrupted Nutrient-Sensing Signals

Accumulating evidence demonstrates that dietary fat modulates nutrient-sensing signals that are responsible for lipid detection, satiation signals, food intake, and weight gain [114,115]. These nutrient-sensing signals include AMP-activated protein kinase (AMPK) [116], sirtuin-1 (SIRT1) [117], PPAR, and PPARγ coactivator-1α (PGC-1α) [118].

In pregnancy, the maternal diet can regulate fetal metabolism and development via nutrient-sensing signals [119]. Accordingly, an imbalanced diet during gestation could disrupt nutrient-sensing signals, having a decisive impact on adult diseases of developmental origins [120,121]. 

Maternal-high-fat-diet-primed hypertension is associated with the inhibitory AMPK/SIRT1/PGC-1α pathway in an offspring’s kidneys [71,75]. AMPK can phosphorylate PGC-1α and regulate its downstream PPARγ signaling. Prior work indicated that specific sets of PPAR target genes participate in renal programming [120]. Although several natural and synthetic PPAR agonists have been studied in kidney-related disorders [46,122,123,124], whether PPAR modulators have protective actions against maternal-high-fat-diet-induced adverse renal outcomes in offspring is awaiting further elucidation.

### 4.5. Gut Microbiota Dysbiosis

The gut microbiome is highly diverse and harbors trillions of microorganisms coexisting with the host, which in turn can determine human health and disease [125]. The shaping and multiplication of gut microbiota start at birth, but the modification of their composition depends on nutritional and environmental factors. Accordingly, maternal dietary nutrients play a key role in the modulation of an offspring’s gut microbiome composition [126]. 

Current evidence suggests that high saturated fat can lower microbiota richness and diversity [127,128]. Similarly, reduced α-diversity in gut microbiota was noted in adult rat offspring from dams fed on a diet rich in fat [129]. In addition, a maternal high-fat diet inducing hypertension in offspring has been linked to an increased *Firmicutes*-to-*Bacteroidetes* (F/B) ratio, which is considered to be a microbial marker for hypertension [130]. Moreover, the reduction in beneficial microbes, a feature of dysbiotic gut microbiota such as *Lactobacillus* and *Akkermansia* [131,132], was reduced in the maternal high-fat diet model [70,74]. 

Microbial metabolites, such as tryptophan-derived metabolites, SCFAs, trimethylamine (TMA), and trimethylamine *N*-oxide (TMAO), are also involved in the pathogenesis of renal programming [133,134,135]. One study indicated that maternal exposure to a high-fat diet could reduce fecal propionate concentration, an SCFA, in 3-week-old rat progeny [74]. Conversely, perinatal propionate supplementation was shown to protect adult offspring born to mother rats with CKD against hypertension [136]. In addition, a maternal high-fat diet increased TMA concentrations and decreased the TMAO-to-TMA ratio [74]. As the inhibition of microbiota-derived metabolites TMA and TMAO is able to attenuate kidney disease [137], targeting the TMA/TMAO pathway as a reprogramming strategy has been studied in different animal models of renal programming [138,139]. Inhibition of TMA formation by 3,3-Dimethyl-1-butanol (DMB) was reported to reduce plasma TMAO levels in mice fed on a Western diet [140]. Our previous research revealed that maternal DMB treatment prevented high-fructose-diet-induced hypertension in adult offspring via regulating the TMA–TMAO metabolic pathway and reshaping the gut microbiome [138]. Another study demonstrated that maternal CKD led to hypertension and renal hypertrophy in 12-week-old male offspring. These adverse renal programming effects can be prevented by maternal iodomethylcholine (an inhibitor of TMA formation) treatment, which coincides with a reduction in TMAO [139]. However, whether high-fat-diet-primed renal programming can be averted in this regard warrants further investigation. 

In experimental and human CKD, the increases in tryptophan-derived uremic toxins from indole and kynurenine pathways participate in the progression of CKD [141,142]. These tryptophan-derived microbial metabolites are endogenous ligands for aryl hydrocarbon receptor (AhR) [143], which can trigger renal inflammation and fibrosis. As high fat intake can activate AhR signaling [144] and that AhR antagonist resveratrol has been associated with the protection of offspring from renal programming [145], more research on the interconnection between a high-fat diet and AhR is required, as they may be a potential reprogramming approach. Together, the findings above suggest that dysbiotic gut microbiota and their derived metabolites might be a probable reason contributing to maternal-high-fat-diet-primed renal programming.

### 4.6. Inflammation

Inflammation has a role in compromised pregnancies and associated complications [146]. The accumulation of T cells, monocytes/macrophages, and T-cell-derived cytokines are involved in the pathogenesis of hypertension [146]. 

Activated T cells are able to secrete cytokines, such as tumor necrosis factor-alpha (TNF-α) and interferon-gamma (IFN-γ), which have been linked to kidney damage and hypertension in pre-clinical models [147]. 

In CKD, the interplay between inflammation and an imbalance of T regulatory cells (Treg) and T helper 17 (TH17) cells has also been related to hypertension [148]. As several tryptophan-derived uremic toxins are ligands for aryl hydrocarbon receptor (AhR) [149], activation of AhR signaling can initiate inflammation through increasing monocyte adhesion, upregulating proinflammatory gene expression, reducing NO bioavailability, and inducing the expression of endothelial adhesion molecules [150]. 

High-fat diets increase free fatty acid uptake and overexpression of fatty acid uptake systems such as the CD36 scavenger receptor, promoting renal inflammation and kidney injury [151]. One study showed that adult rat progeny born to dams exposed to TCDD developed hypertension, which is related to the activation of AhR signaling and induction of TH17-dependent renal inflammation [152]. Although activation of AhR contributes to high-fat-diet-induced vascular dysfunction [153], more research is required to gain a comprehensive insight into whether maternal-high-fat diet-induced renal programming is attributed to the induction of TH17- and AhR-mediated inflammation. 

### 4.7. Others

Considering that maternal-high-fat-diet-related offspring phenotypes are a complex phenomenon, there might be other mechanistic pathways behind renal programming—for example, epigenetic regulation and dysregulation of H_2_S and sodium transporters. We previously found that maternal high-fat diet considerably altered transcriptome in 1-week-old rat offsprings’ kidneys, with females being more sensitive than males [72]. There were 154 upregulated and 97 downregulated genes identified in the kidneys of female offspring. In addition to effects on the kidney, a maternal high-fat diet also causes significant changes in gene expression in the brain [145], placenta [154], and heart [155] in progeny. Whether organ-specific epigenetic regulation may be involved in maternal-high-fat-diet-primed renal programming deserves to be explored further. 

Hydrogen sulfide (H_2_S) is a member of the growing family of gasotransmitters and has emerged as an important signaling molecule in kidney function [156]. Lower H_2_S levels are observed in many renal pathologies, whereas H_2_S-related interventions could be used as a reprogramming approach for DOHaD-related disease [157]. A maternal high-fat diet caused low plasma H_2_S concentrations and renal H_2_S-releasing activity in male rat offspring [73]. Conversely, therapy with perinatal garlic oil, an H_2_S donor, protected offspring from hypertension that was programmed by a maternal high-fat diet, which was connected to the restoration of the H_2_S signaling pathway. 

In addition, high maternal fat consumption increased the protein level or activity of sodium transporter in an offspring’s kidney [60,62,72]. Considering that increased expression/activity of sodium transporters participates in the development of kidney disease and hypertension in various models [158,159], whether maternal-high-fat-diet-induced renal programming can be attributed to inappropriate expression/activity of sodium transporters deserves further clarification.

## 5. Reprogramming Interventions

With a deeper insight into the mechanisms behind maternal-high-fat-diet-induced renal programming, the advances in developing mechanism-targeted strategies hold the potential for reprogramming. Up to now, reprogramming interventions to offset mechanisms governing the developmental programming of kidney disease have covered avoidance of risk factors, lifestyle modification, nutritional supplementation, and pharmacological therapies [160,161]. 

Because of the adverse effects of a maternal diet rich in saturated fats, a universal approach is required to avoid excessive intake of saturated fats during gestation and lactation and avert kidney disease in offspring. However, dietary supplementation with unsaturated fatty acids during gestation and lactation may have beneficial effects on an offspring’s kidney health. 

Feeding pregnant SHRs with a diet enriched with PUFAs during the last week of pregnancy and lactation attenuated the development of hypertension in their male offspring at 16 weeks of age [162]. Another study reported that perinatal omega-3 PUFA supplementation attenuated maternal-high-fat-diet-induced kidney injury and renal programming in female adult offspring [163]. Similarly, supplementing linoleic acid, an omega-6 PUFA, during gestation and lactation can avert offspring hypertension programmed by a maternal high-fat diet [164]. Our previous research also indicated that targeting omega-6 PUFA arachidonic acid can avert maternal-high-fructose-diet-primed renal programming and offspring hypertension [165].

Several other early-life interventions have been utilized as reprogramming approaches to prevent maternal-high-fat-diet-primed renal programming (as listed in Table 1), covering *Limosilactobacillus fermentum* [64], a SIRT1 activator [68], hydralazine [69], long-chain inulin [70], *Lactobacillus casei* [70], resveratrol [71], garlic oil [73], and an AMPK activator [75]. 

Importantly, the gut microbiome is an emerging target for most reprogramming interventions for improving maternal-high-fat-diet-primed renal programming. Probiotics and prebiotics are the most frequently studied gut microbiota-targeted tools. Both have long been acknowledged for their benefits to human health [166,167] and in treating kidney disease [168,169]. Probiotic treatment with *Lactobacillus casei* [70] and *Limosilactobacillus fermentum* [64] during gestation and lactation averts offspring hypertension programmed by high maternal fat consumption. Additionally, prebiotic treatment with long-chain inulin protected offspring from maternal-high-fat-diet-induced renal programming and was related to an increased abundance of beneficial microbe *Lactobacillus* species, increased fecal SCAF concentrations, and reduced plasma TMAO levels [70]. Another study revealed that garlic, a natural prebiotic, offered protection from hypertension to maternal-high-fat-diet-primed offspring, accompanied by increases in α-diversity and abundance of beneficial bacteria *Lactobacillus* and *Bifidobacterium* and plasma SCFA concentrations [73].

As a reprogramming strategy, resveratrol has been utilized to avert maternal-high-fat-diet-primed hypertension in offspring by restoration of the SIRT1/AMPK/PGC1-α pathway [71]. Likewise, the use of SIRT1 activator SRT1720 [68] or direct AMPK activator 5-aminoimidazole-4-carboxamide riboside showed beneficial effects in this regard.

Moreover, a previous study demonstrated that low-dose hydrazine treatment can be beneficial in protecting against renal programming [69]. Hydralazine, a BP-lowering agent with DNA demethylating activities [170], was shown to improve kidney fibrosis at low doses independently of BP [171]. In the maternal high-fat diet model, low-dose hydralazine administration during pregnancy can attenuate albuminuria and glomerulosclerosis, and the increased serum concentration of creatinine in adult offspring is possibly due to epigenetic regulation [69].

Of note is that several interventions targeting mechanistic pathways underlying renal programming have been proven to be effective in averting adult-onset kidney diseases with the utilization of different animal models [6,8,9,12]. These interventions consist of l-cysteine [172], l-citrulline [173], *N*-acetylcysteine [174], melatonin [175], epigallocatechin gallate [176], quabain [177], green tea polyphenol [178], l-carnitine [179], and acetate [180]. Although several early-life interventions have highlighted their potential as an attractive approach to improving kidney health, their efficiency in maternal-high-fat-diet-related renal programming warrants further study. 

## 6. Concluding Remarks

Fats in the maternal diet are like a double-edged sword. Data from preclinical research demonstrate that maternal exposure to a diet enriched with saturated fats is associated with renal programming in adult offspring, including renal function impairment, proteinuria, tubular dysfunction, renal hypertrophy, kidney fibrosis, and hypertension. However, offspring kidney health can be improved via perinatal PUFA supplementation. These findings highlight the importance of a balanced diet during gestation and lactation in determining offsprings’ susceptibility to kidney disease later in life. 

The Kidney Diseases Global Outcomes (KDIGO) 2012 guidelines have not provided specific dietary recommendations for fat intake in patients with CKD [181]. Currently, there is still limited direct evidence linking specific high fat intake in pregnant women and kidney disease in their children. Nevertheless, animal models, such as those described above, provide significant insight into the molecular mechanisms behind maternal-high-fat-diet-primed renal programming. We are fully aware that the presented mechanisms in the present review might not cover the whole picture of the programming actions of high fat consumption. Considering that fats can affect various tissues/organs, consequently leading to different phenotypes in adult offspring, additional research into their organ-specific programming effects is a pressing need. In addition, what is missing from the literature is the comparison of diets with different fat levels and contents on the severity of offspring kidney disease. Hence, it remains difficult to draw a definite conclusion from the available literature based on the wide variations in experimental “high-fat diets” at this time.

Regardless of recent advances in developing potential reprogramming approaches targeting gut microbiota and nutrient-sensing signals for renal programming, almost all of them have not been translated into human trials. In summary, fats in the maternal diet tightly control offspring kidney health and disease. After a greater understanding of maternal-high-fat-induced renal programming, we expect that translating preclinical results into optimal clinical practice is a valuable strategy that could reduce the global burden of kidney disease. 

## Figures and Tables

**Table 1:** Animal models of maternal high-fat-diet-induced renal programming.

Fat Fraction and Component	Species/Gender	Age at Measure (Weeks)	Programming Effects	Refs.
20% (Lard)	SD rat/M & F	52	Reduced renin and Na^+^,K^+^-ATPase activity in kidney	[60]
23% (Saturated fats)	SD rat/M	9	Increased markers of oxidative stress, fibrosis, and inflammation	[61]
24% (Palm oil)	SD rat/M+F	26	Reduced renal Na^+^,K^+^-ATPase activity	[62]
31% (Palm oil)	Wistar rat/M+F	13	Increased lipid peroxidation and reduced SOD activity in the kidneys	[63]
31% (Lard)	Wistar rat/M	14	Increased renal oxidative stress	[64]
34% (mainly linolenic acid and oleic acid)	C57BL/6 mice/M	3	Renal hypertrophy, decreased renal sodium excretion, and increased renal matrix deposition	[65]
35.5% (Lard)	CD-1 mice and GLUT4 heterozygous mice/M	24	Elevated BP and increased renal expression of renin and AT1R	[66]
40% (Saturated fats)	Wistar rat/M	13	Decreased GFR and increased proteinuria	[67]
43% (Saturated fats)	C57BL/6 mice/M	9	Increased renal triglyceride levels, increased renal oxidative stress, inflammatory, and fibrotic markers, as well as increased albuminuria	[68]
43% (Saturated fats)	C57BL/6 mice/M	32	Increased creatinine level, albuminuria, glomerulosclerosis, and renal fibrosis	[69]
58% (Coconut oil)	SD rat/M	16	Elevated BP, increased renal AT1R expression, and alterations in gut microbiota	[70]
58% (Coconut oil)	SD rat/M	16	Elevated BP, decreased urinary NO level, increased renal oxidative stress, and decreased renal Ang-(1–7) level	[71]
58% (Coconut oil)	SD rat/M+F	26	Increased kidney injury and altered renal transcriptome	[72]
58% (Coconut oil)	SD rat/M	16	Elevated BP, dysregulated H_2_S-generating pathway in the kidney, and shifts in gut microbiota composition	[73]
58% (Coconut oil)	SD rat/M	16	Elevated BP, dysregulated nutrient-sensing signals in the kidney, and alterations in gut microbiota composition	[74]
58% (Coconut oil)	SD rat/M	16	Elevated BP and impaired nutrient-sensing pathway in kidneys	[75]

Studies tabulated based on fat fractions in the maternal diet and age at evaluation. SD = Sprague Dawley; GLUT4 = glucose transporter 4; M = male; F = female; NO = nitric oxide; SOD = superoxide dismutase; GFR = glomerular filtration rate; AT1R = angiotensin II type 1 receptor; H_2_S = hydrogen sulfide.

## Data Availability

Data are contained within the article.
